# Finding Suitable Clinical Endpoints for a Potential Treatment of a Rare Genetic Disease: the Case of ARID1B

**DOI:** 10.1007/s13311-020-00868-9

**Published:** 2020-05-22

**Authors:** Matthijs D. Kruizinga, Rob G.J.A. Zuiker, Elif Sali, Marieke L. de Kam, Robert J. Doll, Geert Jan Groeneveld, Gijs W.E. Santen, Adam F. Cohen

**Affiliations:** 1 Centre for Human Drug Research, Zernikedreef 8, 2333 CL Leiden, the Netherlands; 2 Juliana Children’s Hospital, HAGA Teaching Hospital, the Hague, the Netherlands; 3 Department of Clinical Genetics, Leiden University Medical Centre, Leiden, the Netherlands

**Keywords:** ARID1B, Endpoint, Biomarkers, Electrophysiology, Executive functioning, Eye tracking, Cognition

## Abstract

There is a lack of reliable, repeatable, and non-invasive clinical endpoints when investigating treatments for intellectual disability (ID). The aim of this study is to explore a novel approach towards developing new endpoints for neurodevelopmental disorders, in this case for ARID1B-related ID. In this study, twelve subjects with ARID1B-related ID and twelve age-matched controls were included in this observational case–control study. Subjects performed a battery of non-invasive neurobehavioral and neurophysiological assessments on two study days. Test domains included cognition, executive functioning, and eye tracking. Furthermore, several electrophysiological assessments were performed. Subjects wore a smartwatch (Withings® Steel HR) for 6 days. Tests were systematically assessed regarding tolerability, variability, repeatability, difference with control group, and correlation with traditional endpoints. Animal fluency, adaptive tracking, body sway, and smooth pursuit eye movements were assessed as fit-for-purpose regarding all criteria, while physical activity, heart rate, and sleep parameters show promise as well. The event-related potential waveform of the passive oddball and visual evoked potential tasks showed discriminatory ability, but EEG assessments were perceived as extremely burdensome. This approach successfully identified fit-for-purpose candidate endpoints for ARID1B-related ID and possibly for other neurodevelopmental disorders. Next, results could be replicated in different ID populations or the assessments could be included as exploratory endpoint in interventional trials in ARID1B-related ID.

**Electronic supplementary material:**

The online version of this article (10.1007/s13311-020-00868-9) contains supplementary material, which is available to authorized users.

## Introduction

Historically, treatment of intellectual disability (ID) and other neurodevelopmental disorders has focused primarily on the symptoms. Except for a few enzyme deficiency disorders, no treatments of underlying etiology have been incorporated in standard care [1]. Although animal models mimicking clinical ID syndromes have shown promising preclinical data, subsequent trials in humans have failed to show beneficial treatment effects [2, 3]. While translation from mice to humans with ID seems unpredictable, it is generally accepted that a lack of reliable clinical endpoints plays a large role in this disparity. [4] Before conducting further interventional trials in subjects and children with ID, new trial designs and especially endpoints are needed [5]. In this study, we investigate the syndrome ARID1B-related ID as a model to illustrate how to develop new biomarkers for a rare neurodevelopmental disorder.

ARID1B-related ID is caused by haploinsufficiency of *ARID1B*. Pathogenic variants in *ARID1B* have been identified as a cause of Coffin–Siris syndrome in 2012 for the first time [6, 7]. Since then, over 143 patients have been identified and the gene has now been associated with a variable array of phenotypes, ranging from Coffin–Siris syndrome to mild behavioral abnormalities [8]. Most commonly, patients suffer from ID, speech and vision impairment, and (partial) agenesis of the corpus callosum, and display distinct facial features [8, 9]. Mice with *Arid1b* haploinsufficiency showed similar symptoms and were found to have a reduced number of inhibitory GABA-ergic interneurons, causing a presumed inhibition–excitation imbalance which could be partly reversed by the GABA-A positive allosteric modulator clonazepam [10]. Since clonazepam is considered safe, it is a good candidate drug to investigate in patients. However, without fit-for-purpose endpoints, a trial would be doomed to fail.

Central nervous system (CNS) endpoints in ID trials should be considered fit-for-purpose when they satisfy a number of criteria [11]. In our opinion, they must reflect neurological and functional aspects relevant to the disease and be sensitive to detect pharmacological CNS effects. In the case of ID, endpoints should also be non-invasive and easily conducted. Repeatability should be determined in the targeted population and there should be a clear differentiation between patients and control subjects. Ideally, test results should correlate with existing indicators of disease severity.

The NeuroCart® is a neurological test battery known to be sensitive for the detection of CNS effects of compounds [12]. Using this test battery, non-invasive and data-intensive studies can be performed to demonstrate specific, time- and dose-dependent, neurophysiological, and neuropsychological effects [12]. However, the assessments have not been investigated in patients with ID yet. The aim of this study is to explore the characteristics of a battery of non-invasive neurophysiological and neurobehavioral assessments that may be fit-for-purpose as future clinical endpoints for ARID1B-related ID and similar syndromes.

## Materials and Methods

This study was conducted at the Centre of Human Drug Research (CHDR) in Leiden, the Netherlands, from November 2018 until May 2019 and the protocol was reviewed and approved by the Beoordeling Ethiek Biomedisch Onderzoek (BEBO) Foundation Review Board (Assen, the Netherlands). The study was conducted according to the Dutch Act on Medical Research Involving Human Subjects, the Dutch codes of conduct regarding medical research with minors and expression of objection by people with mental disabilities and in compliance with good clinical practice.

### Subjects and Study Design

During this case–control study, twelve patients with pathogenic variants in *ARID1B* were recruited via the Coffin–Siris expertise center of the Leiden University Medical Centre. Twelve age-matched healthy controls were also recruited. Age difference between patients and controls was no more than 2 years, except for adult subjects. Subjects who regularly used benzodiazepines were excluded from the study. Tests were assessed for suitability for two age groups (2–4 and ≥ 5 years old), based on the expected capabilities of the subjects. Tests were performed on two consecutive Saturdays and were repeated 2–4 times during the study. The schedule of assessments is listed in Supplementary Figure S1. Study visits lasted 3–5 h. Baseline characteristics, including the last measured intelligence quotient (IQ) score, were obtained from patient charts. Parents completed the Aberrant Behavior Checklist (ABC) at the start of the study [13].

### Selection of Candidate Endpoints

NeuroCart® tests were selected based on the following criteria: (1) Tests must have demonstrated potential in detecting CNS effects of compounds; (2) Tests must investigate a CNS domain assumed to be affected in patients with ARID1B-related ID; (3) It must be reasonably expected that the tests can be conducted by children and patients with ARID1B-related ID; (4) Ideally, an improvement in test outcomes potentially results in symptom reduction or improvement of quality of life. Selected tests and the accompanying rationale for inclusion are listed in Table 1.

**Table 1:** Rationale for selected tests

	Test	CNS domain	Corresponding ARID1B symptom
Cognition	Animal fluency test	Verbal fluency, semantic memory	Intellectual disability
	VVLT	Memory	Intellectual disability
	Day–night test	Memory and controlled processing	Impulsiveness and intellectual disability
Eye tracking	Smooth pursuit	Attention and oculomotor function	Expected marker for clonazepam effect Expected marker for clonazepam effect
	Saccadic eye movements	Sedation	
Executive functioning	Adaptive tracking	Motor activation and attention	Short attention span
	Finger tapping	Motor activation and fluency	Lethargy and slowness
	Body sway	Balance and attention	Hyperactivity
Electrophysiology	Resting EEG	General CNS activity	Hypothesized abnormal neuronal organization and general CNS functioning Hypothesized abnormal neuronal organization and general CNS functioning
	Passive oddball	Auditory processing	
	Active oddball	Auditory processing	
	VEP	Visual processing	
	ASSR	Auditory processing	
trial@home	Steel HR—physical activity	General daily activity	Hyperactivity, apathy, and lethargy
	Steel HR—sleep parameters	Sleep	Insomnia
	Steel HR—heart rate	Sympathetic activation and arousal	Hyperactivity

VVLT = visual verbal learning test; EEG = electroencephalography; VEP = visual evoked potential; ASSR = auditory steady state response

### Test Procedures

#### Cognition

For the animal fluency test, subjects were asked to verbally produce as many different animals as they could sum up within 60 s [14]. Animals that were named twice or more did not count towards the total amount of animals named and neither were infant versions of adult animals already named. During the visual verbal learning test (VVLT), subjects were presented 30 words in three consecutive word trials [15]. Each trial ended with a free recall of the presented words. Thirty minutes after the first trial, subjects were asked to recall the words. Immediately thereafter, subjects underwent a memory recognition test, consisting of 15 presented words and 15 “distractors.” The day–night task, a simplified version of the Stroop test suitable for children, was included in the study to assess memory and controlled processing of subjects [16].

#### Eye Tracking

Recording of eye movements was performed in a quiet room with dimmed illumination. Analysis was conducted with a microcomputer-based system for sampling of eye movements. Disposable electrodes (Ambu Blue Sensor N) were applied on the forehead and beside the lateral canthi of both eyes. Skin resistance was minimalized before measurements. Head movements were restrained using a fixed head support. Subjects were asked to focus on a moving dot displayed on a computer screen. Saccadic eye movements were recorded for stimulus amplitudes of approximately 15° to either side. Fifteen saccades were recorded with inter-stimulus intervals varying randomly between 3 and 6 s. Average values of saccadic peak velocity (degrees/s) of correct saccades were recorded. At least five detected saccades were necessary to include for statistical analysis. For smooth pursuit eye movements, the target moves sinusoidally at frequencies ranging from 0.3 to 1.1 Hz. Four cycles were recorded for each stimulus frequency. The time during which the eyes were in smooth pursuit of the target was calculated and expressed as a percentage of stimulus duration [17].

#### Executive Functioning Assessments

The adaptive tracking test is a pursuit-tracking task and was performed as described by Borland and Nicholson [18] using customized equipment and software. The subjects were instructed to keep a dot inside a moving circle by operating a joystick. The speed of the moving circle adapted in response to subject performance. After a run-in period of 30 s, the average tracking performance (%) of 3.5 min was used for statistical analysis. The finger tapping test was adapted from the Halstead Reitan Test Battery [19]. Speed of finger tapping was measured for the index finger for the dominant hand; a session contained five performances of 10 s. Subjects were instructed to tap a button as quickly as possible with the index finger of the dominant hand. The mean tapping rate was used for statistical analysis. Body sway was conducted by all subjects and assessed using a pot string meter (Celesco) based on a Wright ataxiameter, with a string attached to the waist. [20] All body movements over 2 min were integrated and expressed as sway in mm. Before starting a measurement, subjects were asked to stand still and comfortable with their hands in a relaxed position. Subjects wore an eye cap to block sight.

#### Electrophysiological Assessments

Complete technical details of measurements and analysis of electrophysiological assessments are listed in Supplementary Text 1. To measure general CNS activity, resting-state EEG with open and blocked eyes was recorded. Spectral analysis of the α1, α2, β1, β2, β3, δ, and θ waves was performed to calculate the power of the respective wavebands at FzCz, PzO1, and PzO2. VEPs (visual evoked potentials) were recorded over the scalp overlying the occipital cortex. During the VEP assessment, a pattern reversal paradigm was used with two phase-changing checkerboards (1.0 and 0.25° pattern). The oddball paradigm is a neuropsychological test to evoke event-related potentials (ERPs). During the passive oddball task, subjects were seated with EEG cap and headphones on and instructed to sit still and relax. Subjects were watching a silent movie while being presented auditory tones as frequent stimuli and infrequent stimuli. For the active oddball task, subjects were to pay attention to the tones and press a button when they heard an infrequent tone. The auditory steady-state response (ASSR) is an electrophysiological response to periodic auditory stimulation [21], thought to be generated through entrainment of neuronal populations to periodic stimuli, and reveal the integrity of neuronal networks. During the test, auditory stimuli with a 500 ms burst of 1 ms monophasic rectangular pulses were presented through headphones. The interstimulus interval was 700 ms. The ASSR was assessed through spectral modulations and inter-trial phase coherence (ITPC).

### trial@home

During the 6 days between measurements, subjects wore a Steel HR watch (Withings, Issy-les-Moulineaux, France), which is incorporated in the CHDR MORE® trial@home platform and which registered step count, heart rate, and several accelerometer-derived sleep parameters. After the final study assessments, parents and children completed a questionnaire regarding the general study experience.

### Statistics

Considering the exploratory nature of this study, no formal power calculation was performed. Statistical analysis was performed with SAS v9.4 (SAS Institute, Cary, NC, USA). The difference between patients and controls was calculated via a repeated-measure mixed-model analysis of variance with fixed factors group, measurement, and group by measurement and subject as random factor. Based on the model, a minimal detectable effect size (MDES) was calculated for a hypothetical crossover study with 16 ARID1B subjects. The MDES was expressed as the proportion of the difference between ARID1B-related ID patients and controls in order to determine whether the effect size is a reasonable goal for future interventional studies. Spearman correlations between mean test outcomes and IQ and ABC subscales were calculated for tests for which a significant difference between patients and controls was demonstrated. Promasys® 7.3 (Omnicomm, Fort Lauderdale, Texas, USA) was used for data management.

### Criteria for Candidate Endpoints

Tests where considered fit-for-purpose when fulfilling the following requirements: (1) Tolerable, meaning subjects showed no signs of resistance during the test; (2) Conducted correctly by the study population, with more than 75% of the outcomes suitable for analysis; (3) Stable over time, defined as a coefficient of variability (CV) within the ARID1B group not higher than 50%; (4) Statistically significant difference between healthy and control subjects; (5) Show an MDES which is less than 50% of the difference between ARID1B subjects and control subjects. Ideally, there is an association between test outcome and IQ or relevant ABC subscales.

## Results

### Baseline Characteristics

A total of 20 parents of patients with ARID1B-related ID were approached, of which 12 consented to participate with their child. Twelve healthy age-matched controls were included. Baseline characteristics are displayed in Table 2. Subjects with ARID1B-related ID scored highest on the hyperactivity, lethargy, and irritability ABC subscales. Parents found the length of study days to be too long (55%), but none indicated they would not participate in a similar study again. The youngest ARID1B subject was 2 years old and found performing tests too difficult, after which the second study day was canceled. There were no adverse events during the conduct of this study. Individual subject characteristics are listed in Supplementary Table S1.

**Table 2:** Baseline characteristics

	ARID1B (*n* = 12)	Controls (*n* = 12)
Age^1^ (mean (range))	12.6 [2–31]	11.8 [2–27]
Sex, female (*n* (%))	9 (75)	12 (100)
Conc. medication (*n* (%))	3 (17)	1 (8)
IQ (mean ± SD)^2^	74 ± 21	–
Can read age appropriately (*n* (%))^3^	8 (67)	12 (100)
Can write age appropriately (*n* (%))^3^	8 (67)	12 (100)
Behavioral problems (*n* (%))^2^	7 (58)	0 (0)
Speech delay or impairment (*n* (%))^2^	12 (100)	0 (0)
Vision problems (*n* (%))^2^	7 (58)	0 (0)
ABC subscale score (mean ± SD)		
Irritability	8.3 ± 7.4	–
Lethargy	11.2 ± 17.2	
Stereotypic behavior	2.4 ± 2.1	
Hyperactivity	13.1 ± 10.0	
Inappropriate speech	1.0 ± 1.5	

^1^The mean age difference between patients and corresponding controls was 0.75 years. ^2^Data obtained from patient charts, when available. ^3^Parent-reported

### Candidate Endpoints

All conducted tests were assessed according to the specified criteria. Summarized results are displayed in Table 3. Individual test performance is displayed in Supplementary Table S2.

**Table 3:**
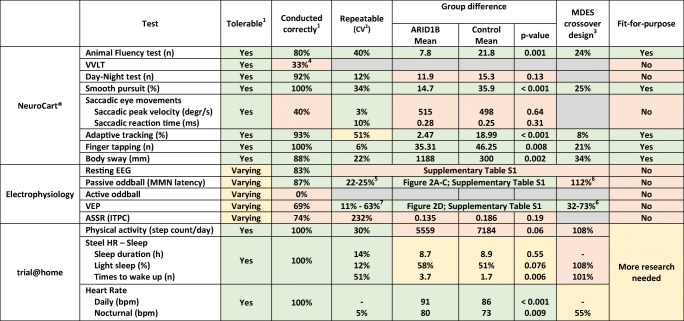
Systematic evaluation of assessments to determine suitability as endpoint in clinical trials

Colors: green: suitable; red: unsuitable; yellow: indeterminate. CV = coefficient of variability, MDES = minimal detectable effect size, VVLT = visual verbal learning test, EEG = electroencephalography, MMN = mismatch negativity, VEP = visual evoked potential, ASSR = auditory steady state response, ITPC = inter-trial phase coherence, bpm = beats per minute

^1^By ARID1B-related ID subjects, investigator’s assessment after exit interview and end-of-study questionnaire with parents; ^2^coefficient of variability within the group of ARID1B-related ID subjects; ^3^minimal detectable effect size, expressed as the proportion of the difference between patients and controls that can be detected as improvement in a crossover study with *n* = 16; ^4^Only 1 of the first 3 ARID1B-related ID subjects was able to obtain a valid score, after which the test was removed from the study protocol; ^5^range of CV of the MMN latency at Cz and Fz; ^6^range of MDES calculated only for parameters with a significant difference between ARID1B subjects and controls; ^7^range of CVs of collected parameters

#### Cognition

**Fig. 1:**
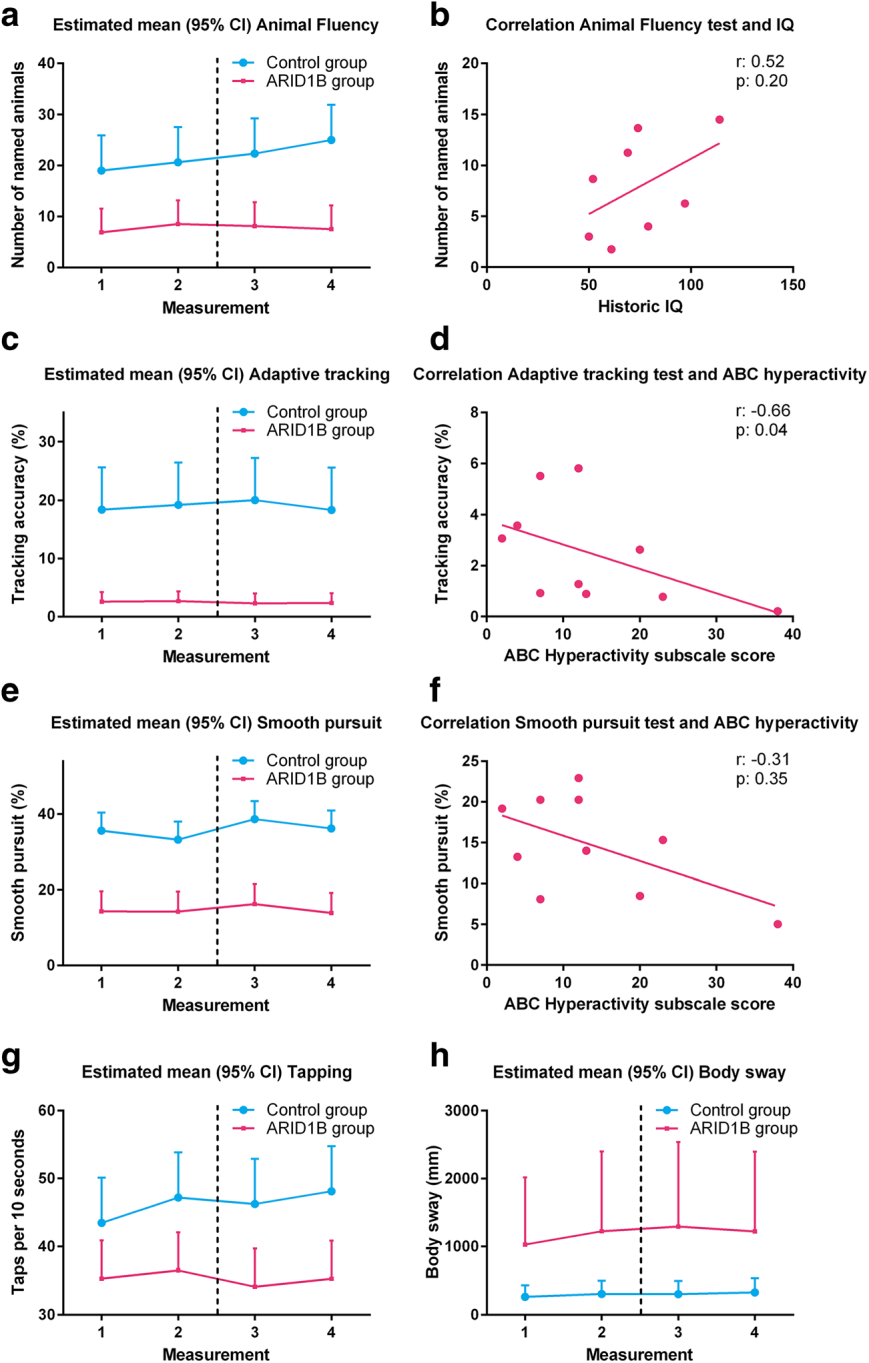
Estimated group means and exploratory correlations of NeuroCart® tests. (A) Mean outcome of the animal fluency test per subject group and measurement number. (B) Linear correlation between historic IQ score and mean animal fluency test score. (C) Mean adaptive tracking test outcome per subject group and measurement number. (D) Linear correlation between the ABC hyperactivity subscale and mean adaptive tracking test score. (E) Mean smooth pursuit eye movement test outcome per subject group and measurement number. (F) Linear correlation between the ABC hyperactivity subscale and mean smooth pursuit eye movement test score. The dotted lines demarcate the two study days

The animal fluency test was successfully conducted by 80% of participants. One nonverbal patient did not complete the test. There was a significant difference between the two study groups and a positive correlation between the number of named animals and IQ (Fig. 1). Day–night test results did not differ between the two study groups (*p* = 0.133). The VVLT was considered too difficult and resulted in stress for the first three patients, after which the test was removed from the study.

#### Executive Functioning and Eye Tracking

All executive functioning tests (adaptive tracking, body sway, finger tapping) were tolerable and conducted correctly. Notably, finger tapping was the favorite assessment for 85% of subjects and 73% of parents. There was a clear and significant difference between subjects and controls for the three tests, while a correlation was present between the ABC hyperactivity subscale and adaptive tracking results. Patients demonstrated a significantly lower smooth pursuit capability compared to controls and there was a correlation between mean smooth pursuit results and the ABC hyperactivity subscale (Fig. 1).

#### Electrophysiological Tests

**Fig. 2:**
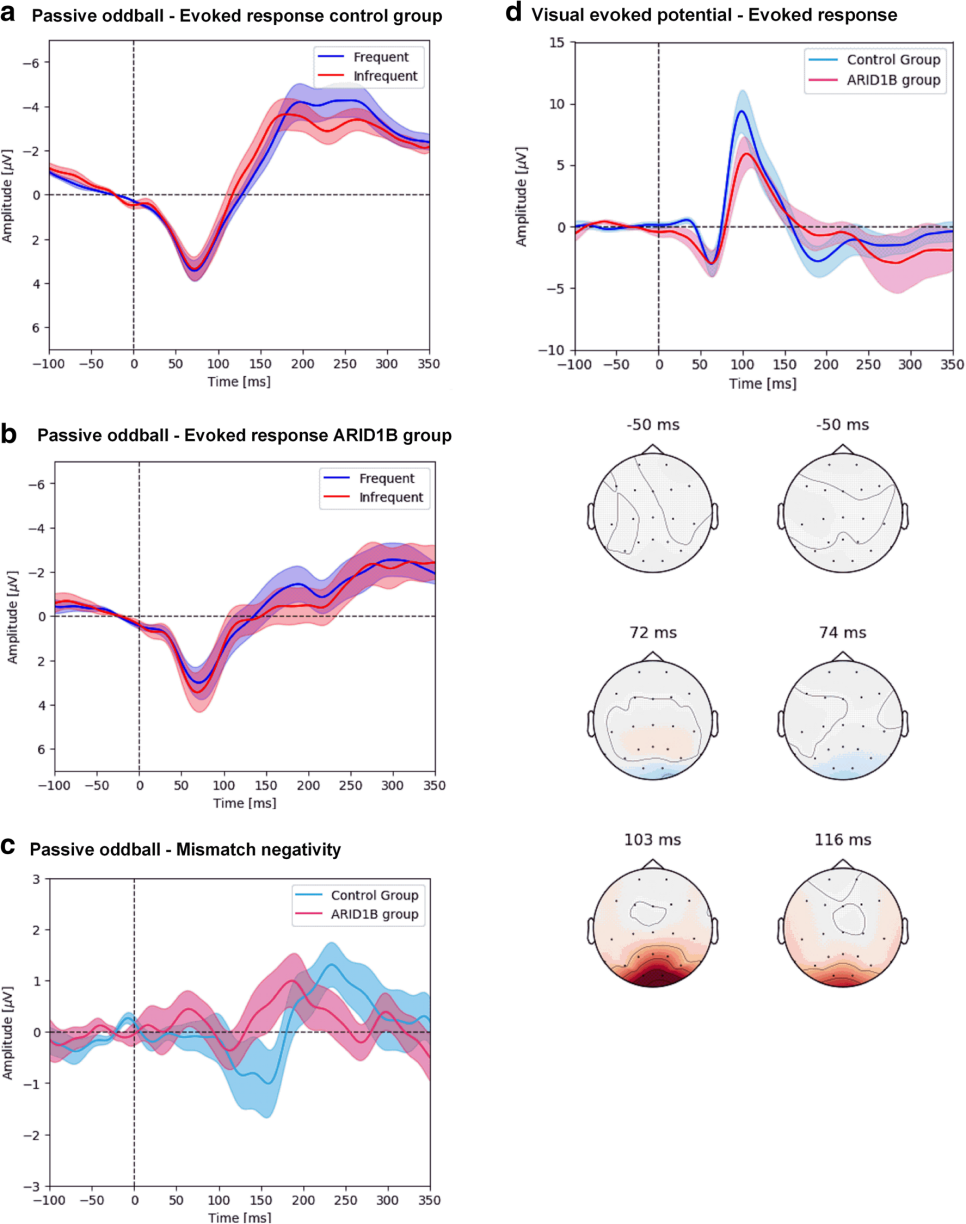
ERPs of patients and controls for passive oddball and VEP assessments. (A) Grand mean of the evoked response during the passive oddball task for ARID1B subjects. (B) Grand mean of the evoked response during the passive oddball task for control group. (C) Mismatch negativity graph. (D) Visual evoked potential (VEP) ERP graph after visual stimulation with 1.0° phase-changing checkerboard, including EEG heat map (left: control group; right: ARID1B group). Although subjects were also stimulated with a 0.25° checkerboard, the high prevalence of refractive ametropia among ARID1B patients in combination with their disability made it impossible to determine whether all subjects saw the 0.25° checkerboard clearly. A statistical summary of passive oddball and VEP analysis are listed in Supplementary Table S1

All 24 subjects completed at least one resting-state EEG. The difference in group means of individual electrodes and EEG parameters are displayed in Supplementary Table S3. On average, a slightly higher α2, δ, and θ power was detected in the occipital electrodes in ARID1B patients compared to healthy controls. The passive oddball ERP graph is displayed in Fig. 2A–C. ARID1B patients had a statistically significant difference in mismatch negativity (MMN) latency at Cz compared to controls (183 ms vs 141 ms, *p* = 0.014), while MMN latency at Fz and the amplitude were statistically similar. The evoked responses were different (Fig. 2A, B). The active oddball paradigm was considered too difficult for the first three patients and was subsequently removed from the study. VEP evoked response (Fig. 2D) demonstrates significantly lower amplitude and longer latency of the P100 peak, as well as a smaller N75-P100 peak-to-peak amplitude. ASSR was performed successfully in 74% of measurements, but there was no significant difference regarding ITPC and evoked power between patients and controls. Fifty percent of patients and controls found setting up the EEG cap to be generally uncomfortable and 42% of parents and 48% of subjects indicated EEG assessments were their least favorite. EEG analysis was suboptimal due to recurrent signal artifacts caused by movements of both ARID1B subjects and the younger control subjects, which led to a low overall signal quality.

#### trial@home

The Steel HR watch was tolerated by all subjects. 73% of subjects and 100% of parents indicated 6 days of measurements was enough or short. There was a difference in physical activity of 1625 steps per day between patients and controls, although this did not reach conventional significance (*p* = 0.06). The sleep duration of both groups was similar (8.7 h for patients and 8.9 h for controls), but patients woke up significantly more often (3.7 vs 1.7, *p* = 0.006). Accordingly, there was a trend towards a lower proportion of light sleep per night and a significantly higher nocturnal heart rate for patients.

## Discussion

In this study, twelve patients with ARID1B-related ID and twelve age-matched controls performed a battery of non-invasive neurophysiological and neurobehavioral assessments. All assessments were reviewed for suitability as new clinical endpoints in clinical trials investigating interventions in populations with (ARID1B-related) ID. This study represents the first of its kind, providing extensive neurobehavioral and neurophysiological phenotypes of a population with an ultra-rare condition.

Of the included tests investigating cognition, the animal fluency test was identified as a promising endpoint fulfilling all predefined criteria. Considering the presence of ID in patients, the difference compared to controls was expected. However, the animal fluency test shows stability over time and the absence of a learning effect in the ARID1B group, making it suitable for the assessment of acute and medium-term treatment effects.

Smooth pursuit eye movements fulfilled the criteria for candidate endpoints as well. In contrast, there was no statistical difference in saccadic peak velocity between patients and controls. We hypothesize this is due to the fact saccadic eye movements are a relatively preserved mechanism involving brainstem responses, while smooth pursuit is a function requiring coordination of multiple brain regions, vulnerable to developmental abnormalities [22, 23]. This has been previously demonstrated in autism and schizophrenia [24, 25]. The difference in the proportion of correctly conducted tests between smooth pursuit and saccadic eye movements was striking. We hypothesize it is more difficult to concentrate on the dot when it randomly changes position during the saccadic eye movement test, as opposed to the continuously moving dot during smooth pursuit. In the future, continuous encouragement during the test may improve the amount of analyzable results.

The three executive functioning tests were found to be suitable candidate endpoints for future studies. This is the first study to investigate these assessments in subjects with ID for this purpose, and overall test results were in line with observed symptoms. For example, patients moved considerably more than controls during the body sway test, reflecting the restlessness that ARID1B subjects exhibit. The patients’ slower finger tapping may express the lethargy patients with ARID1B-related ID suffer from. The adaptive tracking test was also positively assessed on all criteria and was correlated with the ABC hyperactivity subscale. The MDES of the ARID1B group in a crossover study with 16 subjects (1.349), relative to the found difference between the control and ARID1B group (16.52) is 8%, making the adaptive tracking test the most sensitive test within this study to detect potential treatment effects.

We performed a range of electrophysiological assessments investigating general CNS activity and auditory and visual processing. Interpretation of all electrophysiological assessments was hampered due to a poor signal quality. Final analysis was performed after excluding trials of insufficient quality. For the passive oddball paradigm, ARID1B subjects appeared to exhibit a longer latency of the MMN at Cz, but not the amplitude or the latency at Fz. This may reflect an impaired automatic auditory processing ability also found in one other study in subjects with ID [26]. The general evoked response appeared to be smaller for both frequent and infrequent tones (Fig. 2A, B**,**), and the grand mean of the MMN (Fig. 2C) shows two small negative components before and after the average latency. This may be due to unidentified subgroups within ARID1B patients, but displaying grand means of the MMN is not ideal in this study. The MMN matures at increasing age [27]. which leads to varying MMN latencies for the different age groups. In the context of biomarker development, the estimated group means obtained via mixed model analysis may be more suitable. VEP demonstrated a longer latency and lower amplitude of the P100 peak, indicating a slower automatic visual processing ability. The complete electrophysiological substrate of these results goes beyond the scope of this paper.

Several studies have shown that pharmacological activity can alter the ERP waveform, making them an interesting, and potentially non-invasive, biomarker for drug effect in neurological disease [28]. While electrophysiological assessments can theoretically be performed by subjects of all ages, assessments were considered quite invasive for ARID1B subjects. The recurrent movement artifacts caused a significantly reduced data quality, and results should be interpreted with care. These findings show the value of our approach: EEG and ERPs could be very useful biomarkers in clinical trials, but recurring tests are unsuitable in this population. One could even argue that incorporation of EEG assessments in future trials would introduce bias, giving only children who are less affected by the disease the chance to repeatedly perform the assessments.

We demonstrated that an unobtrusive smartwatch can be used for home-monitoring of ID patients. Of the collected parameters, notable differences between patients and controls were found in the nocturnal parameters (number of times to wake up, nocturnal heart rate). There was a trend towards a lower physical activity level per day in patients compared to controls, although this was not a significant difference. We expect that it may be possible to detect adverse or unexpected effects of treatments, such as difficulty sleeping or apathy resulting in a decrease in physical activity using the measurements described here. However, other Withings® smartwatch models have shown a lack of reliability compared to the gold standard regarding measurement of sleep and sleep data should be interpreted with caution [29].

To summarize, we assert that the combination of animal fluency, finger tapping, body sway, adaptive tracking, smooth pursuit eye movements, and possibly home-monitoring with the Steel HR, represents a promising battery of non-invasive tests suitable for interventional studies. In our opinion, this battery of tests is non-invasive and can be conducted correctly by ID patients of 5 years and older. Furthermore, the MDES of tests calculated for a study with a feasible sample size (*n* = 16) reflect reasonable improvements of less than 50% of the difference between patients and controls.

Except for adaptive tracking, we found no statistically significant correlations with the traditional endpoints IQ and ABC subscales. However, significance was not expected considering this study was not powered adequately for this, and the limitations of endpoints currently used in ID trials. IQ and questionnaires have traditionally been used, but IQ has a high intra-subject variability [30], especially at a young age [31], while interpretations of questionnaires are subjective and invariably suffer from inter-rater bias [4]. Objective and standardized tests with low intra-subject variability are more suitable for early phase drug research in small patient groups. Still, improvement in parent-reported behavior certainly represents value for the individual patients and their parents. A combination of objective tests and subjective parent-reported outcomes in future trials may therefore emerge as the best paradigm.

This study has several limitations. First, the recruitment of patients focused on relatively mentally and physically competent ARID1B subjects thought to be able to tolerate traveling to the research location and being administered the test battery. Therefore, the generalizability of the study results regarding patients with severe ID is unclear. We believe the study subjects represent the population that would participate in any interventional clinical trial as well. Several cut-offs, such as for the CV and MDES, were chosen rather arbitrarily and could be more clearly defined when designing a follow-up study. We used historical IQ as a variable to correlate test outcomes with, introducing another factor of uncertainty. Historical IQ of healthy subjects was not available and could therefore not be compared between the groups. However, none of the included control subjects had learning difficulties and historical IQ was only used in the correlation with cognitive test outcomes. Although correlation of raw cognitive scores with age-adjusted standard scores such as IQ is unconventional, this cannot be avoided when no normative values of the included assessments have been determined yet. Age showed some correlation with average test outcome (Supplementary Figure S2), but this was expected and does not negatively impact the fit-for-purpose assessment of the most promising tests. A strength of this study is the repeated measurements design, generating robust data about the variability of study assessments. The included battery of tests investigated all functional CNS domains [12, 32], resulting in a comprehensive neurophysiological and neurobehavioral phenotype of ARID1B-related ID. While many psychometric properties of the candidate endpoints are unknown in the ID population, most tests have been performed in a pediatric population in the past (unpublished data) and have been extensively investigated in adult neurological disorders [12]. While the included tests have at least a theoretical relationship between disease severity and test outcome, as outlined in Table 1, this relationship must be confirmed in future studies. The included healthy controls allowed us to calculate an MDES relative to the control group, which aids in the interpretation of the effect size. Finally, we have included a relatively large cohort of patients considering the total population of patients with ARID1B-related ID in the Netherlands.

This study shows that our approach towards the identification of fit-for-purpose endpoints in rare neurodevelopmental disorders has been successful in the case of ARID1B-related ID. During the next stage of endpoint development, the identified candidate endpoints could be included as exploratory or secondary endpoint in interventional trials in ARID1B-related ID. Furthermore, since there is a large phenotypic variability within the population, test outcomes could be compared in subgroups throughout the ARID1B spectrum. We expect our results are not specific for ARID1B-related ID. Future studies should also focus on the identified potential endpoints in patients with similar syndromes. Furthermore, prior to conducting trials investigating long-term treatment effects over a time span of years, studies aiming to uncover natural progression in test outcomes over time in patients should be performed in order to properly isolate long-term treatment effects during analysis [33].

## Conclusion

We have identified the animal fluency test, adaptive tracking, smooth pursuit eye movement, finger tapping, and body sway as promising endpoints for clinical trials in patients with ARID1B-related ID. More research is needed in the field and physical activity and sleep monitoring. The results from this study will be used in the preparation of an interventional clinical trial investigating the effects of clonazepam in patients with ARID1B-related ID.

## Electronic supplementary material

ESM 1 (PDF 194 kb)

ESM 2 (PDF 286 kb)

ESM 3 (PDF 299 kb)

ESM 4 (PDF 205 kb)

ESM 5 (PDF 215 kb)

ESM 6 (PDF 186 kb)

ESM 7 (PDF 463 kb)

## Data Availability

The data that support the findings of this study are available from the corresponding author upon reasonable request.

## Abbreviations

ABC: Aberrant behavior checklist
ASSR: Auditory steady-state response
ARID1B: AT-rich interaction domain 1B
CHDR: Centre for Human Drug Research
CNS: Central nervous system
CV: Coefficient of variability
EEG: Electroencephalography
ERP: Event-related potentials
ID: Intellectual disability
ITPC: Inter-trial phase coherence
IQ: Intelligence quotient
MDES: Minimal detectable effect size
VEP: Visual evoked potentials
VVLT: Visual verbal learning test
