# Supplementary material for: Finding Suitable Clinical Endpoints for a Potential Treatment of a Rare Genetic Disease: the Case of ARID1B
Source: Neurotherapeutics. 2020 May 22;17(3):1300–10. doi: 10.1007/s13311-020-00868-9 (PMC7609730; doi:10.1007/s13311-020-00868-9)

Supplementary Figure S1 - Schedule of assessments

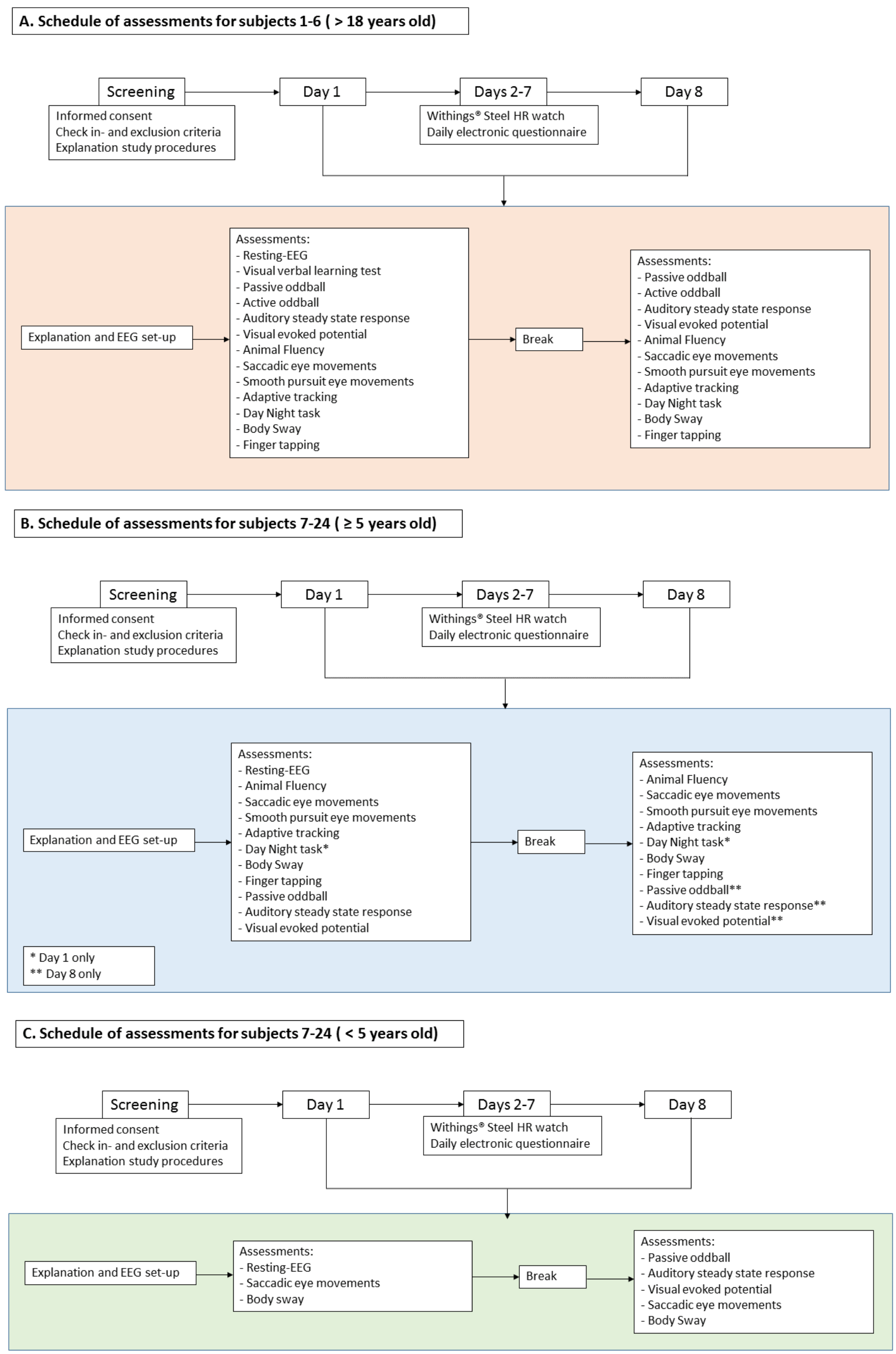

Supplement: Supplementary file 1 — (PDF 194 kb) [file 13311_2020_868_MOESM1_ESM.pdf]
