# Supplementary material for: Finding Suitable Clinical Endpoints for a Potential Treatment of a Rare Genetic Disease: the Case of ARID1B
Source: Neurotherapeutics. 2020 May 22;17(3):1300–10. doi: 10.1007/s13311-020-00868-9 (PMC7609730; doi:10.1007/s13311-020-00868-9)
Supplement: Supplementary file 3 — (PDF 299 kb) [file 13311_2020_868_MOESM3_ESM.pdf]

Supplementary Text 1: Standard operating procedures for EEG and ERP assessments

<u>*Resting EEG*</u>

Task description
Resting-state EEG recordings with open and closed eyes for 5 min in each eye state were performed (Jobert et al., 2012). Each recording employed alternating periods with eyes open and blocked with an eye cap, with a duration of 64-seconds for each period. Subjects faced a featureless wall and were instructed not to stare, not to move their head and eyes, and to try to suppress eye blinks.

Hardware description
EEG was continuously recorded using a 40-channel recording system (Refa-40, TMSi B.V., the Netherlands). Electrodes were placed according to the international 10-20 system (32-lead cap, TMSi B.V.), but replacing electrodes placed at the earlobes (i.e., A1 and A2) with electrodes placed at the mastoids (i.e., M1 and M2). The scalp electrode impedance was kept below 5kΩ. The ground electrode was placed at AFz. Additionally, to detect ocular artefacts, vertical and horizontal EOG was also recorded. Two Ag/AgCl electrodes were placed at the outer canthi of both eyes, and two Ag/AgCl electrodes were placed approximately 2 cm above and below the right eye. All signals were sampled at a sampling rate of 1024 Hz and were filtered prior to storage using a first order recursive high-pass filter with a cut-off frequency at 0.1 Hz. Digital markers were recorded by the amplifier indicating the start and end of each eye state.

Analysis description
Recorded channels were band-pass filtered using a third order Butterworth filter with cut-off frequencies at 0.1 and 45.0 Hz. The filtered signals were then divided into four second epochs. Epochs containing ocular artifacts were removed for further analysis. A power spectrum density (PSD) was calculated for each epoch and averaged for each eye state. The resulting PSDs were then subdivided into bands (see Table 1), and the total power per band is calculated. The electrodes of interest for this study are Fz-Cz, Pz-O1, and Pz-O2.

Table 1. Frequency ranges for spectral analysis

**Band**	**Frequency range [Hz]**
Delta (δ)	1.5 < 6.0
Theta (θ)	6.0 < 8.5
Alpha (α)	8.5 < 12.5
Beta (β)	12.5 < 30.0
Gamma (γ)	30.0 < 40.0

<u>*Passive oddball task*</u>

Task description
Subjects were seated with EEG cap and headphones on and were instructed to sit still and relax. During the task, subjects were watching a silent movie while being presented auditory tones. Subjects were not

to pay special attention to the tones. A total of 750 tones were presented of which 600 presented as frequent stimuli and 150 as deviant/infrequent stimuli. Therefore, infrequent tones had a probability of 0.2. The first five trials were frequent tones and there were at least two frequent tones between two deviant/infrequent tones. The frequent and infrequent tones were 150ms at a sound pressure level of 80dB, respectively. All tones had a 5ms rise and fall time. Tones were presented at a fixed rate of 2 Hz.

Hardware description
EEG was continuously recorded using a 40-channel recording system (Refa-40, TMSi B.V., the Netherlands). Electrodes were placed according to the international 10-20 system (24-lead cap, TMSi B.V.), but replacing electrodes placed at the earlobes (i.e., A1 and A2) with electrodes placed at the mastoids (i.e., M1 and M2). The scalp electrode impedance was kept below 5kΩ. The ground electrode was placed at AFz. Additionally, to detect ocular artefacts, vertical and horizontal EOG were also recorded. Two Ag/AgCl electrodes are placed at the outer canthi of both eyes, and two Ag/AgCl electrodes were placed approximately 2 cm above and below the right eye. All signals were sampled at a sampling rate of 1024 Hz and are filtered prior to storage using a first order recursive high-pass filter with a cut-off frequency at 0.1 Hz. Digital markers were recorded by the amplifier the onset of auditory stimuli.

Analysis description
Recorded channels were bandpass filtered between 1 and 30 Hz using a 2nd order two-pass reverse Butterworth filter. Channels were then visually inspected and parts containing large muscle, movement, or technical artefacts were removed from the analysis. Artefacts caused by ocular movements were corrected for using Blind Source Separation techniques (either Independent Component Analysis [ICA] or Second Order Blind Identification [SOBI]).

Channels were then re-referenced to the average mastoids. The continuous data was then segmented into 500ms epochs: from -50ms to +450ms. The average of all frequent epochs was subtracted from the average of infrequent/deviant epochs resulting in the mis-match negativity waveform. The MMN-amplitude and latency were then defined as the maximum amplitude and latency of the maximum amplitude of the difference waveform between 100 and 250ms post-stimulus, respectively. The amplitude and latency were determined at Fz and Cz.

<u>Active oddball task</u>

Task description
Subjects were seated with EEG cap and headphones on and were instructed to sit still and relax. During the task, subjects were being presented auditory tones. Subjects were asked to pay attention to the tones and press a response-button when they heard an infrequent/deviant tone. A total of 500 tones were presented of which 400 as frequent stimuli and 100 as deviant/infrequent stimuli. Therefore, infrequent tones had a probability of 0.2. The first five trials were frequent tones and there were at least two frequent tones between two deviant/infrequent tones. The frequent and infrequent tones were 150ms tones of 1000 Hz and 500 Hz at a sound pressure level of 75dB, respectively. All tones had a 5ms rise and fall time. Tones were presented at a fixed rate of 1 Hz.

Hardware description
EEG was continuously recorded using a 40-channel recording system (Refa-40, TMSi B.V., the Netherlands). Electrodes were placed according to the international 10-20 system (24-lead cap, TMSi B.V.), but replacing electrodes placed at the earlobes (i.e., A1 and A2) with electrodes placed at the

mastoids (i.e., M1 and M2). The scalp electrode impedance was kept below 5kΩ. The ground electrode was placed at AFz. Additionally, to detect ocular artefacts, vertical and horizontal EOG were also recorded. Two Ag/AgCl electrodes were placed at the outer canthi of both eyes, and two Ag/AgCl electrodes were placed approximately 2 cm above and below the right eye. All signals were sampled at a sampling rate of 1024 Hz and filtered prior to storage using a first order recursive high-pass filter with a cut-off frequency at 0.1 Hz. Digital markers were recorded by the amplifier the onset of auditory stimuli.

Analysis description
Recorded channels were low-pass filtered with a cut-off frequency of 30 Hz using a 2nd order two-pass reverse Butterworth filter. Channels were then visually inspected and parts containing large muscle, movement, or technical artefacts are removed from the analysis. Artefacts caused by ocular movements were corrected for using Blind Source Separation techniques (either Independent Component Analysis [ICA] or Second Order Blind Identification [SOBI]).

Channels were then re-referenced to the average mastoids. The continuous data was then segmented into 1000ms epochs: from -150ms to +850ms. The P300-amplitude and latency were then defined as the maximum amplitude and latency of the maximum amplitude of the deviant-waveform between 280 and 420ms post-stimulus, respectively. The amplitude and latency were determined at Fz, Cz, and Pz.

Auditory steady state response

Task description
Auditory Steady State Response (ASSR) recordings were performed. Subjects were seated in a comfortable chair and instructed to sit still, relax, and to close their eyes. They were also instructed to not pay special attention to auditory tones. Auditory stimuli were presented through headphones at a sound pressure level of 65 dB+- 2 dB. Each stimulus was a 500ms burst of 1ms monophasic rectangular pulses at 40 Hz. The inter-stimulus interval was 700ms with no onset asynchrony.

EEG recording
EEG was continuously recorded using a 40-channel recording system (Refa-40, TMSi B.V., the Netherlands). Electrodes were placed according to the international 10-20 system with a total of 21 leads (MFi B.V.), but replacing electrodes placed at the earlobes (i.e., A1 and A2) with electrodes placed at the mastoids (i.e., M1 and M2). The scalp electrode impedance was kept below 5kΩ. The ground electrode was placed at AFz. Additionally, to detect ocular artefacts, vertical and horizontal EOG were recorded. Two Ag/AgCl electrodes were placed at the outer canthi of both eyes, and two Ag/AgCl electrodes were placed approximately 2 cm above and below the right eye. All signals were sampled at a sampling rate of 1024 Hz and are filtered prior to storage using a first order recursive high-pass filter with a cut-off frequency at 0.1 Hz. Digital markers were recorded by the amplifier indicating the onset of each stimulus.

Analysis
Recorded channels were band-pass filtered with cut-off frequencies at 1.0 and 48.0 Hz. A Blind Source Separation technique was used to correct for ocular artefacts. Single trial epochs were extracted from -150ms to +600ms relative to stimulus onset. EEG channels and epochs were visually inspected and those containing large muscle, movement, or technical artefacts were removed for further analysis. Epochs were then re-referenced to the average mastoid channels. All further analysis focused on Fz.

For each recording, the inter-trial phase coherence (ITPC) was calculated using a Morlet wavelet transform. The average ITPC between 35 and 45 Hz, and between 200 and 500ms was used as the endpoint. Evoked power was based on the square of the real part of a Hilbert transform on the average of all trials. The pre-stimulus average between -150 and 50ms are subtracted from the endpoints for both the ITPC and evoked power.

<u>Visual evoked potentials</u>

Task description
The VEPs (Visual Evoked Potentials) are electrical potentials evoked by brief visual stimuli (or sharp changes in visual stimuli) recorded over the scalp overlying the occipital cortex. Here, we focus on pattern-reversal ERPs, which typically exhibit characteristic N75, P100 and N135 peaks. VEPs are sensitive to alterations present along the visual pathway (occipital cortex, optic nerve, optic radiations, retina or eye) (Odom et al., 2016) and therefore affected by many conditions.

The task was designed following the standard for clinical visual evoked potentials issued by the International Society for Clinical Electrophysiology of Vision (Odom et al., 2016). Subjects are seated with an EEG cap and are instructed to sit still, place their head in the chin-rest and focus on the red cross on the screen.

While watching the red cross on the screen, subjects are presented phase changing checkerboard-patterns. The phase of checkerboards change every 500ms. There are two different checkerboard sizes: 1.00 degree and 0.25 degree (see Figure 2 for an illustrative example). Each checkerboard size changes phase a total of 320 times. Between the two types of stimuli a short break for subjects is allowed. Details of the task parameters are described in

Table 2. The total task duration is about 8 min.

Analysis description
Analysis of the EEG recordings were performed by the (Senior) Clinical Research Engineer after all recordings of the study were collected. Recorded channels were bandpass filtered between 1 and 30 Hz, either using a 500th order FIR filter or a 2nd order two-pass reverse Butterworth filter. Channels were then visually inspected and parts containing large muscle, movement, or technical artefacts were removed from the analysis. Artefacts caused by ocular movements were corrected for using Blind Source Separation techniques (either Independent Component Analysis [ICA] or Second Order Blind Identification [SOBI]).

All channels were re-referenced to Fz. The continuous data was then segmented into 500ms epochs: from -100 to 400ms relative to the onset of the phase reversals. The first 20 phase reversals for each checkerboard size were rejected for analysis as these were intended for familiarizing subjects to the stimuli. The VEP was then calculated by averaging over all trials on either Oz or, if unavailable, the average of O1 and O2. The following peaks and corresponding latencies were then automatically determined:

- N75: minimum value and latency between 29 and 114 ms
- P100: maximum value and latency between 86 and 143 ms

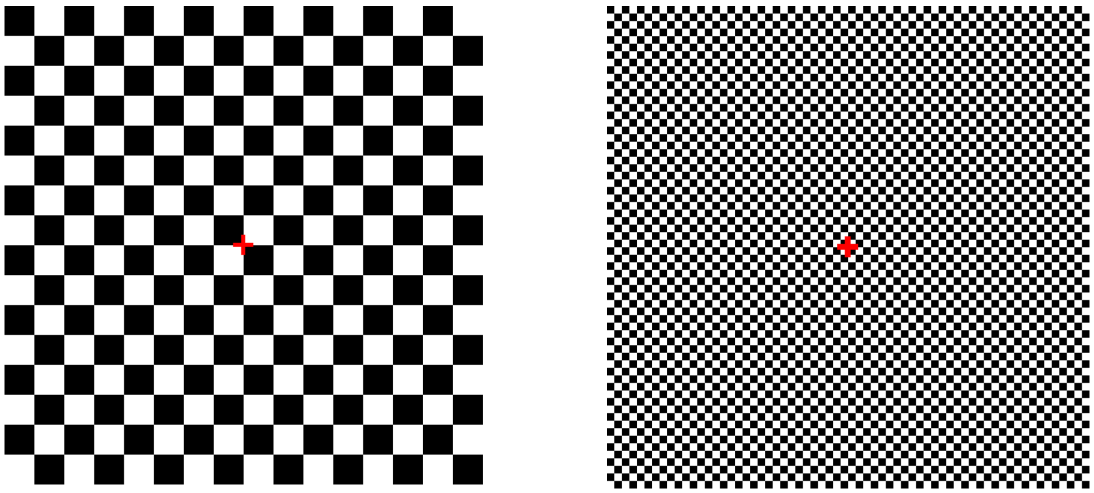

A B

Figure 2 Two illustrative examples of checkerboard. (A) Checkerboard with a relatively large angle between blocks. (B) Checkerboard with a relatively small angle between blocks.

Table 2. VEP Task parameters

Parameter	Specifications
Presentation	Binocular
Presentation rate	2 reversals per second
Distance subject to screen	58cm
Width of checks	1.00 degrees (Figure A) and 0.25degrees (Figure B)
Total width of the checkerboard	15.6 degrees
Michelson contrast	Larger than 80 %
Mean luminance	50 $cd.m^{-2}$ +/- 2.5cd/m^2

References

Odom JV, Bach M, Brigell M, Holder GE, McCulloch DL, Mizota A, Tormene AP (2016) ISCEV standard for clinical visual evoked potentials—(2016 update). Doc Ophthalmol 133(1):1–9
