# Supplementary material for: Finding Suitable Clinical Endpoints for a Potential Treatment of a Rare Genetic Disease: the Case of ARID1B
Source: Neurotherapeutics. 2020 May 22;17(3):1300–10. doi: 10.1007/s13311-020-00868-9 (PMC7609730; doi:10.1007/s13311-020-00868-9)
Supplement: Supplementary file 4 — (PDF 205 kb) [file 13311_2020_868_MOESM4_ESM.pdf]

Supplementary table S1 – Individual clinical characteristics

Subject	Age	IQ[1]	ARID1B-ID or ARID1B-CSS[2]	cDNA	Protein	Behavioural problems	Speech delay or impairment	Can read	Can Write	Hearing loss	Vison impairment	History of epilepsy	Agenesis of corpus callosum
**1**	2	NA	CSS	c.2692C>T	p.Arg898*	NA	Yes	No	No	No	Yes	No	Hypoplasia
**2**	3	NA	ID	c.5404C>T	p.Arg1802*	Yes	Yes	No	No	No	No	Yes	Yes
**3**	5	97	ID	c.1160_1200del	p.Ala387Glyfs*134	No	Yes	Yes	Yes	Yes	Yes	No	Unknown
**4**	6	114	CSS	c.2572_2573delinsCTGG	p.Ala858Leufs*57	NA	Yes	Yes	Yes	No	No	No	Partial
**5**	7	79	ID	c.5074del	p.leu1691fs	Yes	Yes	No	No	No	No	No	No
**6**	10	74	ID	c.1960C>T	p.Gln654*	Yes	Yes	Yes	Yes	No	No	No	Unknown
**7**	11	69	ID	c.1579C>T	p.Gln527*	No	Yes	Yes	Yes	No	Yes	No	Unknown
**8**	14	52	ID	c.6511C>T	p.Gln2171*	Yes	Yes	Yes	Yes	Yes	No	No	No
**9**	18	50	ID	c.2510dup	p.Tyr.840*	Yes	Yes	No	No	No	Yes	No	Yes
**10**	22	61	CSS	c.3223C>T	p.Arg1075*	No	Yes	Yes	Yes	No	Yes	No	Partial
**11**	22	56	CSS	c.2318C>G	p.Ser773*	Yes	Yes	Yes	Yes	Yes	Yes	No	Yes
**12**	31	92	ID	c.2917dup	p.Met973Asnfs*16	Yes	Yes	Yes	Yes	No	Yes	Unknown	Unknown

[1] Historical IQ obtained from patient charts. In the case of a range given (e.g. 54-58, the mean was used for statistical and graphical purposes.)
[2] ARID1B-CSS: a priori Coffin-Siris syndrome diagnosis before the mutation was identified, ARID1B-ID: the mutation was identified without an a priori diagnosis. Usually through exome sequencing or micro-array investigations.
