# Supplementary material for: Finding Suitable Clinical Endpoints for a Potential Treatment of a Rare Genetic Disease: the Case of ARID1B
Source: Neurotherapeutics. 2020 May 22;17(3):1300–10. doi: 10.1007/s13311-020-00868-9 (PMC7609730; doi:10.1007/s13311-020-00868-9)
Supplement: Supplementary file 5 — (PDF 215 kb) [file 13311_2020_868_MOESM5_ESM.pdf]

Supplementary table 2 – Individual subject characteristics and successfully performed tests

Subject	Age	IQ*	Animal fluency test	VVLT	Day-night test	Smooth pursuit eye movements	Saccadic eye movements	Adaptive tracking	Finger tapping	Body sway	Resting EEG	Active oddball	Passive oddball	ASSR	VEP	Trial@ home
**1**	2															
**2**	3															
**3**	5	97														
**4**	6	114														
**5**	7	79														
**6**	10	74														
**7**	11	69														
**8**	14	52														
**9**	18	50														
**10**	22	61														
**11**	22	56														
**12**	31	92														

Colors: gray = NA; green = performed successfully 100% of the time; yellow = performed successfully > 50% of the time, red = performed successfully < 50% of the time

* Historical IQ obtained from patient charts. In the case of a range given (e.g. 54-58, the mean was used for statistical and graphical purposes.)
