# Supplementary material for: Finding Suitable Clinical Endpoints for a Potential Treatment of a Rare Genetic Disease: the Case of ARID1B
Source: Neurotherapeutics. 2020 May 22;17(3):1300–10. doi: 10.1007/s13311-020-00868-9 (PMC7609730; doi:10.1007/s13311-020-00868-9)
Supplement: Supplementary file 6 — (PDF 186 kb) [file 13311_2020_868_MOESM6_ESM.pdf]

Supplementary Table S3 – Statistical summary of individual EEG and ERP parameters

		LS Means			Contrasts (95% CI) p-value
	Parameter	Control group	ARID1B group	Treatment P-value	ARID1B group Control group
Resting EEG	EEG Alpha1-power Fz-Cz: eyes closed ((uV)^2)	8.3385	8.9904	0.7538	7.817% (-34.1%, 76.29%) p=0.7538
	EEG Alpha1-power Fz-Cz: eyes open ((uV)^2)	5.9413	7.4514	0.4628	25.42% (-33.1%, 135.2%) p=0.4628
	EEG Alpha1-power Pz-O1: eyes closed ((uV)^2)	36.5526	38.1726	0.8892	4.432% (-44.9%, 97.93%) p=0.8892
	EEG Alpha1-power Pz-O1: eyes open ((uV)^2)	12.4966	19.7419	0.1885	57.98% (-21.5%, 218.1%) p=0.1885
	EEG Alpha1-power Pz-O2: eyes closed ((uV)^2)	42.5913	42.8384	0.9866	.5802% (-50.4%, 104.2%) p=0.9866
	EEG Alpha1-power Pz-O2: eyes open ((uV)^2)	14.7881	20.7852	0.3623	40.55% (-34.3%, 200.8%) p=0.3623
	EEG Alpha2-power Fz-Cz: eyes closed ((uV)^2)	4.1766	3.2711	0.3120	-21.7% (-52.0%, 27.79%) p=0.3120
	EEG Alpha2-power Fz-Cz: eyes open ((uV)^2)	2.7287	3.1284	0.5473	14.65% (-28.1%, 82.73%) p=0.5473
	EEG Alpha2-power Pz-O1: eyes closed ((uV)^2)	11.0408	12.8167	0.5762	16.09% (-32.8%, 100.6%) p=0.5762
	EEG Alpha2-power Pz-O1: eyes open ((uV)^2)	5.5235	10.5542	0.0031	91.08% (27.67%, 186.0%) p=0.0031
	EEG Alpha2-power Pz-O2: eyes closed ((uV)^2)	12.3525	14.0603	0.6384	13.83% (-35.6%, 101.3%) p=0.6384
	EEG Alpha2-power Pz-O2: eyes open ((uV)^2)	6.3724	10.7510	0.0146	68.71% (12.06%, 154.0%) p=0.0146
	EEG Alpha-power Fz-Cz: eyes closed ((uV)^2)	13.6041	12.4692	0.6587	-8.34% (-38.9%, 37.46%) p=0.6587
	EEG Alpha-power Fz-Cz: eyes open ((uV)^2)	9.1525	10.8809	0.4920	18.88% (-28.9%, 98.75%) p=0.4920
	EEG Alpha-power Pz-O1: eyes closed ((uV)^2)	53.7718	54.1579	0.9774	.7182% (-40.1%, 69.44%) p=0.9774
	EEG Alpha-power Pz-O1: eyes open ((uV)^2)	19.1059	31.7865	0.0611	66.37% (-2.56%, 184.1%) p=0.0611

	Parameter	LS Means		Treatment P-value	Contrasts (95% CI) p-value
		Control group	ARID1B group		ARID1B group Control group
Resting EEG	EEG Alpha-power Pz-O2: eyes closed ((uV)^2)	62.6376	60.0181	0.8807	-4.18% (-46.6%, 71.92%) p=0.8807
	EEG Alpha-power Pz-O2: eyes open ((uV)^2)	22.6964	33.5377	0.1699	47.77% (-16.5%, 161.6%) p=0.1699
	EEG Beta1-power Fz-Cz: eyes closed ((uV)^2)	3.8496	4.4925	0.5571	16.70% (-32.4%, 101.4%) p=0.5571
	EEG Beta1-power Fz-Cz: eyes open ((uV)^2)	3.4094	4.1823	0.4343	22.67% (-28.3%, 109.9%) p=0.4343
	EEG Beta1-power Pz-O1: eyes closed ((uV)^2)	9.0167	13.5012	0.0563	49.74% (-1.18%, 126.9%) p=0.0563
	EEG Beta1-power Pz-O1: eyes open ((uV)^2)	6.8105	10.0894	0.0669	48.15% (-2.95%, 126.1%) p=0.0669
	EEG Beta1-power Pz-O2: eyes closed ((uV)^2)	9.9469	13.6178	0.1526	36.91% (-11.9%, 112.8%) p=0.1526
	EEG Beta1-power Pz-O2: eyes open ((uV)^2)	7.5143	10.8415	0.0901	44.28% (-6.07%, 121.6%) p=0.0901
	EEG Beta2-power Fz-Cz: eyes closed ((uV)^2)	1.1249	0.8375	0.1804	-25.5% (-52.3%, 16.30%) p=0.1804
	EEG Beta2-power Fz-Cz: eyes open ((uV)^2)	1.0491	0.8109	0.2632	-22.7% (-51.6%, 23.35%) p=0.2632
	EEG Beta2-power Pz-O1: eyes closed ((uV)^2)	2.6920	2.2832	0.4677	-15.2% (-46.6%, 34.78%) p=0.4677
	EEG Beta2-power Pz-O1: eyes open ((uV)^2)	1.7270	2.0021	0.5304	15.93% (-28.3%, 87.52%) p=0.5304
	EEG Beta2-power Pz-O2: eyes closed ((uV)^2)	2.9184	2.4526	0.4531	-16.0% (-47.8%, 35.27%) p=0.4531
	EEG Beta2-power Pz-O2: eyes open ((uV)^2)	1.9253	2.1334	0.6541	10.81% (-30.7%, 77.19%) p=0.6541
	EEG Beta3-power Fz-Cz: eyes closed ((uV)^2)	2.4635	1.7150	0.1020	-30.4% (-55.2%, 8.256%) p=0.1020
	EEG Beta3-power Fz-Cz: eyes open ((uV)^2)	2.2456	1.7067	0.2303	-24.0% (-52.1%, 20.64%) p=0.2303
	EEG Beta3-power Pz-O1: eyes closed ((uV)^2)	4.6378	4.4977	0.9134	-3.02% (-45.8%, 73.56%) p=0.9134

	Parameter	LS Means		Treatment P-value	Contrasts (95% CI) p-value
		Control group	ARID1B group		ARID1B group Control group
Resting EEG	EEG Beta3-power Pz-O1: eyes open ((uV)^2)	3.8749	4.6443	0.5497	19.85% (-35.5%, 122.7%) p=0.5497
	EEG Beta3-power Pz-O2: eyes closed ((uV)^2)	5.0743	5.6062	0.7599	10.48% (-43.7%, 116.8%) p=0.7599
	EEG Beta3-power Pz-O2: eyes open ((uV)^2)	4.2169	4.9268	0.6199	16.84% (-38.7%, 122.6%) p=0.6199
	EEG Beta-power Fz-Cz: eyes closed ((uV)^2)	7.5993	7.1940	0.8101	-5.33% (-41.2%, 52.51%) p=0.8101
	EEG Beta-power Fz-Cz: eyes open ((uV)^2)	6.8444	6.8420	0.9988	-.034% (-38.0%, 61.09%) p=0.9988
	EEG Beta-power Pz-O1: eyes closed ((uV)^2)	16.8515	21.1171	0.2985	25.31% (-19.4%, 94.87%) p=0.2985
	EEG Beta-power Pz-O1: eyes open ((uV)^2)	12.6811	17.6784	0.1566	39.41% (-12.9%, 123.0%) p=0.1566
	EEG BEta-power Pz-O2: eyes closed ((uV)^2)	18.6388	22.9645	0.3680	23.21% (-23.3%, 97.93%) p=0.3680
	EEG BEta-power Pz-O2: eyes open ((uV)^2)	13.9923	19.0162	0.1869	35.90% (-14.9%, 117.0%) p=0.1869
	EEG Delta-power Fz-Cz: eyes closed ((uV)^2)	35.9416	51.5792	0.3999	43.51% (-40.1%, 243.7%) p=0.3999
	EEG Delta-power Fz-Cz: eyes open ((uV)^2)	31.6461	51.4655	0.2396	62.63% (-29.5%, 275.0%) p=0.2396
	EEG Delta-power Pz-O1: eyes closed ((uV)^2)	43.1354	125.4000	0.0512	190.7% (-.583%, 750.1%) p=0.0512
	EEG Delta-power Pz-O1: eyes open ((uV)^2)	27.9842	96.7561	0.0126	245.8% (34.05%, 791.8%) p=0.0126
	EEG Delta-power Pz-O2: eyes closed ((uV)^2)	42.9019	127.7262	0.0426	197.7% (4.064%, 751.7%) p=0.0426
	EEG Delta-power Pz-O2: eyes open ((uV)^2)	29.5499	104.8099	0.0106	254.7% (38.56%, 807.9%) p=0.0106
	EEG Gamma-power Fz-Cz: eyes closed ((uV)^2)	0.7847	0.6115	0.2718	-22.1% (-50.8%, 23.54%) p=0.2718
	EEG Gamma-power Fz-Cz: eyes open ((uV)^2)	0.7609	0.6241	0.3901	-18.0% (-48.8%, 31.47%) p=0.3901

	Parameter	LS Means		Treatment P-value	Contrasts (95% CI) p-value
		Control group	ARID1B group		ARID1B group Control group
Resting EEG	EEG Gamma-power Pz-O1: eyes closed ((uV)^2)	2.0138	2.1506	0.8733	6.796% (-54.3%, 149.6%) p=0.8733
	EEG Gamma-power Pz-O1: eyes open ((uV)^2)	2.0351	2.2543	0.7872	10.77% (-49.1%, 140.9%) p=0.7872
	EEG Gamma-power Pz-O2: eyes closed ((uV)^2)	2.1629	2.5408	0.7178	17.47% (-53.0%, 193.7%) p=0.7178
	EEG Gamma-power Pz-O2: eyes open ((uV)^2)	2.3713	2.4346	0.9497	2.671% (-56.5%, 142.2%) p=0.9497
	EEG Theta-power Fz-Cz: eyes closed ((uV)^2)	10.6278	32.0562	0.0351	201.6% (8.949%, 735.1%) p=0.0351
	EEG Theta-power Fz-Cz: eyes open ((uV)^2)	8.5865	27.9236	0.0185	225.2% (24.65%, 748.4%) p=0.0185
	EEG Theta-power Pz-O1: eyes closed ((uV)^2)	22.7781	53.0557	0.0916	132.9% (-13.8%, 529.6%) p=0.0916
	EEG Theta-power Pz-O1: eyes open ((uV)^2)	10.2375	29.6572	0.0156	189.7% (24.84%, 572.2%) p=0.0156
	EEG Theta-power Pz-O2: eyes closed ((uV)^2)	22.8577	54.5136	0.0893	138.5% (-13.5%, 557.5%) p=0.0893
	EEG Theta-power Pz-O2: eyes open ((uV)^2)	10.8431	32.2539	0.0150	197.5% (26.38%, 600.1%) p=0.0150
Passive oddball	The average MMN amplitude at Cz (uV)	-1.995	-1.482	0.5332	0.514 (-1.230, 2.257) p=0.5332
	The average MMN latency Cz (ms)	141.310	183.875	0.0144	42.565 (9.404, 75.727) p=0.0144
	The average MMN amplitude at Fz (uV)	-2.533	-2.039	0.4768	0.494 (-0.955, 1.942) p=0.4768
	The average MMN latency at Fz (ms)	162.740	177.419	0.4335	14.679 (-23.777, 53.134) p=0.4335
Visual evoked potential	N75 amplitude 1 deg checkerboard (uV)	-4.734	-2.539	0.1547	2.195 (-0.960, 5.350) p=0.1547
	Peak to peak amplitude N75-P100 1 deg checkerboard (uV)	16.728	8.383	0.0009	-8.345 (-12.751, -3.938) p=0.0009
	P100 amplitude 1 degree checkerboard (uV)	11.947	5.761	0.0060	-6.186 (-10.284, -2.088) p=0.0060

		LS Means			Contrasts (95% CI) p-value
	**Parameter**	**Control group**	**ARID1B group**	**Treatment P-value**	**ARID1B group Control group**
Visual evoked potential	N75 amplitude 0.25 degree checkerboard (uV)	-8.210	-2.634	0.0004	5.576 (2.861, 8.292) p=0.0004
	Peak to peak amplitude N75-P100 0.25 deg checkerboard (uV)	16.114	4.656	<.0001	-11.458 (-15.739, -7.178) p=<.0001
	P100 amplitude 0.25 deg checkerboard (uV)	7.916	1.961	0.0021	-5.955 (-9.425, -2.486) p=0.0021
	Latency N75 peak 1 deg checkerboard (ms)	60.870	59.151	0.6988	-1.719 (-11.130, 7.692) p=0.6988
	Latency P100 peak 1 deg checkerboard (ms)	99.016	114.747	0.0021	15.731 (7.193, 24.270) p=0.0021
	Latency N75 peak 0.25 deg checkerboard (ms)	70.247	64.747	0.3508	-5.500 (-17.568, 6.567) p=0.3508
	Latency P100 peak 0.25 deg checkerboard (ms)	105.114	125.072	0.0008	19.959 (10.611, 29.307) p=0.0008
Auditory steady state response	Evoked power 35-45 Hz and 200-500 ms	0.307	0.402	0.7234	0.095 (-0.461, 0.650) p=0.7234
	Inter-trial phase coherence 35-45 Hz and 200-500 ms	0.186	0.135	0.1847	-0.052 (-0.130, 0.027) p=0.1847
